# The Extracellular Matrix in the Evolution of Cortical Development and Folding

**DOI:** 10.3389/fcell.2020.604448

**Published:** 2020-12-03

**Authors:** Salma Amin, Víctor Borrell

**Affiliations:** Instituto de Neurociencias, Consejo Superior de Investigaciones Científicas and Universidad Miguel Hernández, Sant Joan d’Alacant, Spain

**Keywords:** radial glia, gene expression, microenvironment, folding, evolutionary conservation, extracellular matrix

## Abstract

The evolution of the mammalian cerebral cortex leading to humans involved a remarkable sophistication of developmental mechanisms. Specific adaptations of progenitor cell proliferation and neuronal migration mechanisms have been proposed to play major roles in this evolution of neocortical development. One of the central elements influencing neocortex development is the extracellular matrix (ECM). The ECM provides both a structural framework during tissue formation and to present signaling molecules to cells, which directly influences cell behavior and movement. Here we review recent advances in the understanding of the role of ECM molecules on progenitor cell proliferation and neuronal migration, and how these contribute to cerebral cortex expansion and folding. We discuss how transcriptomic studies in human, ferret and mouse identify components of ECM as being candidate key players in cortex expansion during development and evolution. Then we focus on recent functional studies showing that ECM components regulate cortical progenitor cell proliferation, neuron migration and the mechanical properties of the developing cortex. Finally, we discuss how these features differ between lissencephalic and gyrencephalic species, and how the molecular evolution of ECM components and their expression profiles may have been fundamental in the emergence and evolution of cortex folding across mammalian phylogeny.

## Introduction

The largest part of our brain is the cerebral cortex, or neocortex, which is considered the seat for our higher cognitive abilities and complex reasoning. The extraordinary size and complexity of the human cerebral cortex are the result of a sophisticated and exquisitely orchestrated developmental program, which emerged during mammalian evolution. This stemmed from an increase in the number of neuronal and glial cells, followed by a dramatic expansion in cortical size and folding. The selective pressure on these traits was the basis for the evolution of the mammalian cortex towards human (Florio and Huttner, 2014; De Juan Romero and Borrell, 2015). Recent efforts in understanding this remarkable process of mammalian cortex evolution have begun to shed light on key cellular and molecular mechanisms involved.

The neocortex is a large sheet of neural tissue characteristically organized in six main layers of neurons. This sheet may be smooth, typical of mammals with small brains like mice, or three-dimensionally arranged in folds and fissures, typical of mammals with a large brain like primates and carnivores, including human (De Juan Romero et al., 2015; Fernandez et al., 2016). The cerebral cortex originally develops from the early telencephalic primordium, a pseudostratified epithelium with apical-basal polarity composed by neuroepithelial cells (NECs; Götz and Huttner, 2005; Taverna et al., 2014). Cortical neurogenesis begins with the transformation of NECs into apical Radial Glia Cells (aRGCs), the lineage of which gives rise to all excitatory neurons of the neocortex. aRGCs are highly polarized and elongated cells, with an apical process contacting the ventricular surface, a basal process contacting the pial surface, and the cell body in the vicinity of the telencephalic ventricle, which altogether constitute the ventricular zone (VZ; Boulder_Committee, 1970). Similar to NECs, the cell body of aRGCs migrates apico-basally during the distinct phases of the cell cycle, in a movement known as interkinetic nuclear migration (INM). After mitosis at the apical surface, the cell nucleus moves basally during G1, undergoes DNA replication (S phase) at the basal side of the VZ, and moves apically during G2 to undergo mitosis again at the apical surface (Takahashi et al., 1993). aRGCs typically express the paired-box transcription factor Pax6, and may produce neurons either directly upon mitosis, or indirectly via producing Basal Progenitors (BPs; Noctor et al., 2001, 2004; Haubensak et al., 2004; Miyata et al., 2004). BPs generated by aRGCs migrate to the basal border of the VZ, where they coalesce forming the subventricular zone (SVZ) and divide to eventually produce neurons. There are two main types of BPs: intermediate progenitor cells (IPCs), which lack obvious polarity and characteristically express the T-box transcription factor Tbr2; basal radial glia cells (bRGCs), similar to aRGCs with a basal process contacting the pial surface, but without an apical process contacting the ventricle (Haubensak et al., 2004; Miyata et al., 2004; Noctor et al., 2004; Fietz et al., 2010; Hansen et al., 2010; Reillo et al., 2011; Shitamukai et al., 2011). In species with a smooth cortex (lissencephalic) like mouse, the SVZ is relatively thin and contains few BPs, with IPCs being the predominant type. These BPs largely undergo self-consuming neurogenic divisions, producing two neurons each. In contrast, in species with a folded cortex (gyrencephalic), the SVZ contains much larger numbers of BPs and is much thicker, displaying two cytoarchitectonically distinct sublayers: inner (ISVZ) and outer subventricular zone (OSVZ; Smart et al., 2002; Reillo et al., 2011). The high abundance of BPs in gyrencephalic species is largely due to their high potential for self-amplification (Fietz et al., 2010; Hansen et al., 2010; Betizeau et al., 2013). Both ISVZ and OSVZ are rich in bRGCs and IPCs, which after several rounds of self-amplification start producing massive numbers of neurons (Reillo et al., 2011; Betizeau et al., 2013; Martínez-Martínez et al., 2016). Neurogenesis from BPs occurs either by asymmetric self-renewing divisions (producing one neuron and one progenitor), or by terminal symmetric self-consuming divisions (producing two neurons). Thus, the abundance of BPs is ultimately proportional to the final number of cortical neurons and to cortical folding, these parameters being low in lissencephalic and high in gyrencephalic species (Borrell and Reillo, 2012; Betizeau et al., 2013; Pilz et al., 2013; Dehay et al., 2015; Llinares-Benadero and Borrell, 2019).

The extracellular matrix (ECM) is a key part of the cellular microenvironment during cortical development, contributing to define the local niche of the different cell populations. The ECM is formed by a complex combination of structural proteins and proteoglycans that act as a cell-supporting scaffold. However, in addition to this classical concept, recent studies show that the ECM plays fundamental roles in the polarity, survival, proliferation, migration and differentiation of cells (Hynes, 2009). Recent major breakthroughs in transcriptomic and functional analysis of cortical development in both lissencephalic and gyrencephalic species have identified ECM components as key factors regulating the proliferation of specific types of cortical progenitors, with a direct impact on the expansion and folding of the cerebral cortex (Fietz et al., 2012; Florio and Huttner, 2014; Florio et al., 2017; Long et al., 2018; Long and Huttner, 2019).

Here, we review how the expression of ECM components is regulated and patterned during cortical development, across cortical layers and progenitor cell populations, in lissencephalic and gyrencephalic species. Then we elaborate on the impact of the ECM on cortical progenitor cell proliferation and neuronal migration across mammalian phylogeny, and discuss its influence on the mechanical properties of cortical tissue, altogether affecting cortex folding. Finally, we hypothesize that the modification of ECM components and their expression patterns may have been critical to the remarkable expansion and folding of the mammalian neocortex during evolution.

## Expression of ECM Components During Cortical Development

Transcriptomic analyses of the developing human, mouse and ferret neocortex have been key to our understanding of the relevance of ECM in cortical development (Fietz et al., 2010, 2012; Camp et al., 2015; De Juan Romero et al., 2015; Florio et al., 2015; Pollen et al., 2015; Martínez-Martínez et al., 2016; Telley et al., 2019). High-throughput bulk RNA sequencing (RNAseq) analyses of isolated cortical germinal layers in mouse and human at mid-neurogenesis highlight that specific sets of ECM components are differentially expressed (Fietz et al., 2012). In human embryos, cortical germinal zones including VZ, ISVZ and OSVZ exhibit higher mRNA expression levels of ECM components and cytoskeletal proteins than the neuronal layer Cortical Plate (CP; Table 1). The mouse VZ also has a distinct signature of ECM gene expression, such that these genes are downregulated when progenitor cells are undergoing neurogenesis (Arai et al., 2011). Transcriptomic microarray data from the ferret neocortical VZ also revealed differential expression of ECM components, in this case along cortical developmental stages (Table 1; Martínez-Martínez et al., 2016).

**TABLE 1:** Differentially expressed extracellular matrix (ECM) components, Integrins, growth factors, and transferases, across lissencephalic, and gyrencephalic species.

ECM Genes		Human NCBI Gene ID	A Human (Fietz et al., 2012)			Mouse (Fietz et al., 2012)		B Ferret (Martínez-Martínez et al., 2016)			C Human cell populations (Florio et al., 2015)		
			hVZ	hISVZ = hOSVZ	hCP	mVZ	mCP	E34VZ-E30VZ	P1VZ-E34VZ	P1VZ-E30VZ	aRG > bRG > N	bRG ≥ aRG > N
Proteoglycans	ACAN	176	−	−	−	−	−	nr	nr	nr		ACAN
	BCAN	63827	−	−	−	−	−	BCAN	BCAN	BCAN	−	−
	BGN	633		BGN				nr	nr	nr	−	−
	DCN	1634					DCN	−	−	DCN	−	−
	HAPLN1	1404	−	−	−	−	−	nr	nr	nr	HAPLN1	
	HAPLN4	404037	HAPLN4					nr	nr	nr	−	−
	NCAN	1463	NCAN					−	NCAN	NCAN	−	−
	LUM	4060	−	−	−	−	−	nr	nr	nr	LUM	
	RELN	5649	−	−	−	−	−	−	−	RELN	−	−
	SCUBE3	222663					SCUBE3	−	−	SCUBE3	−	−
	SPARC	6678	−	−	−	−	−	−	SPARC	−	−	−
	SPARCL1	8404	−	−	−	−	−	−	SPARCL1	−	−	−
	SPOCK1	6695	−	−	−	−	−	−	SPOCK1	SPOCK1	−	−
	SPOCK2	9806	−	−	−	−	−	−	SPOCK2	SPOCK2	−	−
	SUSD1	64420	−	−	−	−	−	−	−	SUSD1	−	−
	VCAN	1462					VCAN	−	VCAN	VCAN	−	−
ECM proteins	ATRN	8455	−	−	−	−	−	−	ATRN	ATRN	−	−
	BMPER	168667				BMPER		nr	nr	nr		BMPER
	CD248	57124		CD248				nr	nr	nr	−	−
	CNTN4	152330			CNTN4			nr	nr	nr	−	−
	COCH	1690			COCH			nr	nr	nr	−	−
	ECM1	1893				ECM1		nr	nr	nr	−	−
	FBLN2	2199				FBLN2		−	FBLN2	FBLN2	−	−
	FBLN5	10516	FBLN5					nr	nr	nr	−	−
	LGALS3	3958	−	−	−	−	−	−	−	LGALS3	−	−
	LGALS8	3964			LGALS8			nr	nr	nr	−	−
	LGALSL	29094	−	−	−	−	−	−	LGALSL	LGALSL	−	−
	LTBP1	4052	−	−	−	−	−	−	LTBP1	−	−	−
	LTBP4	8425	−	−	−	−	−	−	LTBP4	LTBP4	−	−
	MATN2	4147	MATN2					−	MATN2	MATN2	−	−
	MFAP1	4236	−	−	−	−	−	−	MFAP1	MFAP1	−	−
	NTN1	9423		NTN1				nr	nr	nr	−	−
	NTN3	4917					NTN3	nr	nr	nr	−	−
	NTN4	59277				NTN4		nr	nr	nr	−	−
	NTNG1	22854	−	−	−	−	−	−	−	NTNG1	−	−
	PRELP	5549	−	−	−	−	−	nr	nr	nr	PRELP	
	RELN	5649	−	−	−	−	−	−	−	RELN	−	−
	TMEFF2	23671			TMEFF2			nr	nr	nr	−	−
	VIT	5212	−	−		−	−	−	−	VIT	−	−
	VWF	7450			VWF			nr	nr	nr	−	−
Collagens	COL1A1	1277	−	−	−	−	−	−	−	COL1A1	−	−
	COL2A1	1280	COL2A1					−	−	COL2A1	−	−
	COL1A2	1278	−	−	−	−	−	nr	nr	nr	COL1A2	
	COL3A1	1281	−	−	−	−	−	−	−	COL3A1	−	−
	COL4A1	1282		COL4A1				−	COL4A1	COL4A1		COL4A1
	COL4A2	1284		COL4A2				nr	nr	nr	−	−
	COL4A6	1288	−	−	−	−	−	−	−	COL4A6	−	−
	COL5A2	1290	−	−	−	−	−	−	−	COL5A2	−	−
	COL5A3	50509	COL5A3					nr	nr	nr	−	−
	COL8A1	1295	−	−	−	−	−	nr	nr	nr		COL8A1
	COL9A3	1299		COL9A3				nr	nr	nr	−	−
	COL11A1	1301	−	−	−	−	−	−	−	COL11A1	−	−
	COL11A2	1302	COL11A2					nr	nr	nr	−	−
	COL12A1	1303				COL12A1		nr	nr	nr	−	−
	COL15A1	1306				COL15A1		−	−	COL15A1	−	−
	COL16A1	1307	−	−	−	−	−	−	COL16A1	COL16A1	−	−
	COL17A1	1308	−	−	−	−	−	−	COL17A1	COL17A1	−	−
	COL18A1	80781				COL18A1		−	COL18A1	COL18A1	−	−
	COL21A1	81578	−	−	−	−	−	COL21A1	COL21A1	COL21A1	−	−
	COL22A1	169044	COL22A1					nr	nr	nr	−	−
	COL24A1	255631	−	−	−	−	−	−	COL24A1	COL24A1	−	−
	COL28A1	340267	−	−	−	−	−	nr	nr	nr	COL28A1	
	COLQ	8292	COLQ					nr	nr	nr	−	−
Laminins	LAMA1	284217	−	−	−	−	−	−	−	LAMA1	−	−
	LAMA3	3909	LAMA3					nr	nr	nr	−	−
	LAMA5	3911				LAMA5		nr	nr	nr	−	−
	LAMB1	3912	−	−	−	−	−	−	LAMB1	LAMB1	−	−
	LAMB2	3913	−	−	−	−	−	−	−	LAMB2	−	−
	LAMB4	22798	−	−	−	−	−	nr	nr	nr		LAMB4
	LAMC2	3918	−	−	−	−	−	nr	nr	nr		LAMC2
Integrins	ITGA1	3672		ITGA1				nr	nr	nr	−	−
	ITGA3	3675			ITGA3			nr	nr	nr	−	−
	ITGA5	3678				ITGA5		nr	nr	nr	−	−
	ITGA10	8515				ITGA10		nr	nr	nr	−	−
	ITGB5	3693	−	−	−	−	−	ITGB5	−	−	−	−
Growth Factors	BMP3	651					BMP3	nr	nr	nr	−	−
	CRELD1	78987	−	−	−	−	−	−	CRELD1	−	−	−
	EREG	2069	−	−	−	−	−	nr	nr	nr		EREG
	FGF5	2250	−	−	−	−	−	nr	nr	nr		FGF5
	FGF9	2254	−	−	−	−	−	FGF9	−	−	−	−
	FGF12	2257			FGF12			nr	nr	nr	−	−
	FGF18	8817					FGF18	nr	nr	nr	−	−
	GDF1	2657					GDF1	nr	nr	nr	−	−
	GDF5	8200					GDF5	nr	nr	nr	−	−
	IGF2	3481	IGF2					nr	nr	nr	−	−
	INHA	3623					INHA	nr	nr	nr	−	−
	INHBA	3624			INHBA			nr	nr	nr	−	−
	MEGF6	1953		MEGF6				nr	nr	nr	−	−
	MEGF8	1954	−	−	−	−	−	−	−	MEGF8	−	−
	MEGF10	84466	−	−	−	−	−	−	MEGF10	−	−	−
	MSTN	2660	MSTN					nr	nr	nr	−	−
	PDGFA	5154			PDGFA			nr	nr	nr	−	−
	PDGFB	5155			PDGFB			nr	nr	nr	−	−
	PDGFC	56034				PDGFC		nr	nr	nr	−	−
	PDGFRA	5156			PDGFRA			nr	nr	nr	−	−
	TGFA	7039				TGFA		nr	nr	nr	−	−
	TGFB3	7043				TGFB3		nr	nr	nr	−	−
	TMEFF2	23671			TMEFF2			−	−	TMEFF2	−	−
	VEGFC	7424				VEGFC		nr	nr	nr	−	−
Transferase	CHPF	79586	CHPF					nr	nr	nr	−	−
	CHSY3	337876			CHSY3			nr	nr	nr	−	−
	HS2ST1	9653				HS2ST1		nr	nr	nr	−	−
	HS6ST1	9394					HS6ST1	nr	nr	nr	−	−
	NDST1	3340				NDST1		nr	nr	nr	−	−
	NDST2	8509				NDST2		nr	nr	nr	−	−
	ST3GAL2	6483			ST3GAL2			nr	nr	nr	−	−
	SULF1	23213	SULF1					nr	nr	nr	−	−
	SULT1B1	27284	−	−	−	−	−	nr	nr	nr		SULT1B1
	SULT1C2	6819	−	−	−	−	−	nr	nr	nr		SULT1C2
	SULT1C4	27233	−	−	−	−	−	nr	nr	nr	SULT1C4	

***(A)** Genes differentially expressed between cortical layers in human and mouse (Fietz et al., 2012). The gene name is indicated where it is expressed at significantly higher levels compared to the other layers; (−) means no significant difference. **(B)** Genes differentially expressed between embryonic (E) and postnatal (P) cortical Ventricular Zone (VZ) in ferret (Martínez-Martínez et al., 2016). The gene name is indicated where it is differentially expressed; (−), no significant difference; (nr), not reported. **(C)** Genes differentially expressed between specific cell populations of the developing human cortex (Florio et al., 2015). The gene name is indicated in the comparison where it is differentially expressed; (−), no significant difference*

Extracellular matrix components are extraordinarily diverse, and many of those expressed in the developing cerebral cortex are polyvalent in regulating stem cell proliferation and niche maintenance (Fietz et al., 2010; Marthiens et al., 2010; Stenzel et al., 2014; Güven et al., 2020). Each mammalian species expresses in cortical germinal zones a unique combination of ECM components at unique relative levels, which suggests that their precise abundance and overall combined composition may be important in fine-tuning cortical progenitor proliferation, self-renewal and expansion, which are also unique among species. In the human OSVZ, very rich in highly proliferative BPs, specific ECM components are expressed at high levels (Table 1). A landmark study by Florio et al. (2015) compared the transcriptomic profile of isolated aRGCs, bRGCs and neurons in the developing human and mouse cerebral cortex. This analysis revealed that ECM components and cell surface receptors were more highly expressed in human aRGCs and bRGCs than in mouse, pointing to the notion that these components may influence the proliferation of aRGCs and bRGCs in human versus mouse (Florio et al., 2015, 2016, 2017). Hence, a notion emerges that each species, either lissencephalic or gyrencephalic, elaborates its own ECM niche in germinal zones to implement the particular proliferative and neurogenic program for their unique set of progenitor cell composition, thus contributing to species differences in cortical development. Accordingly, changes in the expression of ECM components strongly regulate cortical progenitor proliferation and may have been central in the evolutionary expansion of the human neocortex (Fietz et al., 2012). Importantly, germinal zones appear to be a reservoir of ECM components. For example, HAPLN1 and collagen I mRNAs are expressed at high levels in human germinal zones (Table 1), but at the protein level these are concentrated in the CP and cortical wall. This shows that germinal zones are the site of transcription of these genes, but the proteins they encode are only active at the CP and cortical wall (Long et al., 2018).

One of the most salient features of mammalian cortex evolution is its folding. Transcriptomic studies in ferret have shed light on the genetic basis of cortex folding, which also appears to be strongly influenced by the ECM. By comparing the transcriptomic profile of the cortical germinal zones prospectively forming the Splenial Gyrus and the Lateral Sulcus in the ferret visual cortex, we discovered a large number of genes differentially expressed between these two regions, including genes that encode for cell adhesion molecules and ECM components (De Juan Romero et al., 2015). This analysis also showed that the largest amount of differentially expressed genes, and the greatest differences in expression levels between prospective gyrus and sulcus, occur at the OSVZ, further supporting the central importance of this germinal layer in the differential expansion and folding of the cerebral cortex. This pioneer notion has been substantiated experimentally by, for example, the disruption of Integrin receptor function in the OSVZ of ferret organotypic cortical slices (Fietz et al., 2010). The loss of function of Integrin αvβ3 caused a significant reduction in the abundance of bRGCs, but not IPCs. This indicates that ECM components specifically enhance the amplification of bRGCs and, consequently, promote the expansion of the OSVZ and cortex folding (Fietz et al., 2010; De Juan Romero et al., 2015; Dehay et al., 2015).

Single cell RNA sequencing (scRNAseq) revolutionized the field of transcriptomic analysis by providing a snapshot of cell diversity. scRNAseq has been extensively used to characterize the developing cerebral cortex in a variety of mammals, from mouse to human, and newly emerged *in vitro* experimental models such as cerebral organoids (Camp et al., 2015; Pollen et al., 2015; Arlotta and Pasca, 2019; Kanton et al., 2019; Telley et al., 2019; Bhaduri et al., 2020). Aiming to identify the transcriptomic changes that caused the evolutionary expansion of the neocortex, studies have compared aRGCs and bRGCs in human and mouse. Findings highlight ECM genes as a correlate with the high proliferative activity of RGCs in human and ferret as compared to mouse (Lui et al., 2014; Johnson et al., 2015; Pollen et al., 2015). For example, human bRGCs have higher expression levels of ECM genes than mouse, including Laminin, Tenascins, and Integrins, along with HOPX, PTPRZ1, and other genes that modulate the interaction between ECM components, self-renewal of progenitor cells and migration of neurons (Pollen et al., 2015). ScRNAseq analyses have also revealed that RGCs possess unique typological and temporal transcriptomic profiles, distinguishing lineages between the dorsoventral and the rostrocaudal telencephalon. Accordingly, the well-known topographic differences and gradients of development in the telencephalon have been proposed to result from the existence of spatially patterned transcriptomic programs (Nowakowski et al., 2017). Similarly, during development of the mouse somatosensory cortex aRGCs gradually switch from proliferation to neurogenesis, and this appears to be evolutionarily conserved, as it is largely recapitulated in embryonic human aRGCs (Telley et al., 2019). This temporal and spatial change in the transcriptomic profile of progenitor cells during cortical development is linked to ECM components and microenvironmental cues, suggesting that they may have a relevant impact on neurogenesis and cortical patterning.

Recently, cerebral organoids have emerged as a valid *in vitro* model to study cortical development in diverse species (Lancaster et al., 2013; Lancaster and Knoblich, 2014; Camp et al., 2015; Qian et al., 2019; Velasco et al., 2019; Bhaduri et al., 2020). Accordingly, scRNAseq studies comparing progenitor cell populations in human fetal tissue and cerebral organoids have shown that aRGC populations express similar ECM components in both systems (Camp et al., 2015). Interestingly, scRNAseq in human and chimpanzee organoids uncovered subtle differences in the expression levels of genes encoding ECM components and cell adhesion molecules. Given the relevance of differences between human and chimpanzee to understand human evolution, even these small variations in the transcriptomic profiles and signaling pathways of cortical progenitor cells may be key in understanding the evolution and expansion of the human brain (Pollen et al., 2015, 2019; Mora-Bermudez et al., 2016).

## ECM and Proliferation of Neural Progenitor Cells

**FIGURE 1:**
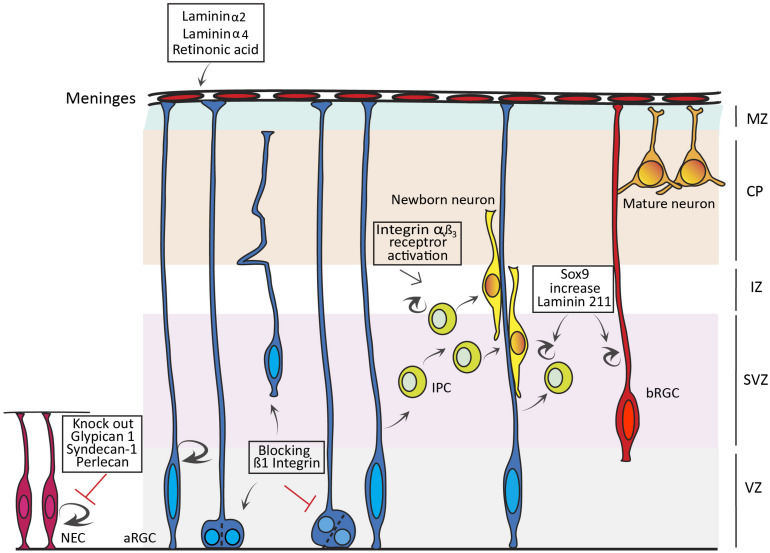
Influence of extracellular matrix (ECM) on cortical progenitor cells. Schema summarizing the effects of ECM components on the proliferation and lineage of neuroepithelial cells (NEC), apical Radial Glia Cells (aRGC) and basal Progenitor Cells (bPC), including Intermediate Progenitor Cells (IPC) and basal RGCs (bRGC). Loss of Glypican 1, Syndecan-1, and Perlecan leads to a decrease in proliferation of NECs, while blocking β1 Integrin leads to apical detachment of aRGCs and loss of asymmetric divisions in the VZ. Knocking out Laminin α2, Laminin α4, and Retinoic acid secreted from the external meninges affects aRGC attachment to the basement membrane. Activation of Integrin αvß3 increases IPC proliferation and cell cycle re-entry, while Sox9 activation increases bPC proliferation via Laminin 211.

The ECM plays many roles during neural development, from the formation of a meshwork for structural support, to the activation of signaling pathways that stimulate progenitor proliferation, either directly or indirectly (Barros et al., 2011). Prior to the onset of neurogenesis, NECs in the cortical primordium augment their number by self-amplification via symmetric divisions (Miyata et al., 2010; Fernandez et al., 2016). Already at that early stage, the ECM provides the microenvironment necessary to modulate the behavior of NECs (Perris and Perissinotto, 2000; Zimmermann and Dours-Zimmermann, 2008). The developing cortex exhibits high concentration of extracellular matrix molecules, including chondroitin sulfate (CS) and heparan sulfate (HS) proteoglycans, hyaluronic acid (HA), Laminins, and glycoproteins like Tenascins (Maeda, 2015). Proteoglycans have an influential role on the proliferation of NECs. These are complex macromolecules composed of a central core with sulphated glycosaminoglycan (GAG) and O- or N-oligosaccharides covalently linked. There are four types of GAGs: CS, dermatan sulfate (DS), Heparin and HS; Schwartz and Domowicz, 2018). Heparan sulfate proteoglycans (HSPGs) include Syndecans, Glypicans, Agrin, and Perlecan (Sarrazin et al., 2011). Glypican is abundant in the cortical VZ during neurogenesis. Mouse embryos mutant for Glypican 1 have an imbalance between proliferation and differentiation of NECs during one day of embryonic development (E8.5-9.5), which is sufficient to cause a significant reduction in brain size (Figure 1). At the signaling level, this reduction is due to the suppression of fibroblast growth factor signaling (FGF; Jen et al., 2009). The evolutionary conservation of the role of Glypican on NECs, and its relationship with FGF signaling, is evident in *Drosophila*, where it has been linked to organ development (Crickmore and Mann, 2007), and in *Xenopus* embryos, where Glypican 4 regulates dorsal forebrain development via FGF signaling activation (Galli et al., 2003).

Perlecan is an ECM component of the basement membrane important for both structural support and NEC proliferation (Figure 1). Mouse embryos mutant for Perlecan exhibit either exencephaly or microcephaly, the latter caused by a reduction in progenitor cell proliferation and impaired cell cycle progression. This phenotype results from a reduced dispersion of growth factors in the extracellular space mediated by Perlecan, such as FGF or SHH (Girós et al., 2007). Perlecan is also highly conserved, where the mutation of its *Drosophila* homolog *trol* leads to G1 cell cycle arrest, mediated by FGF and hedgehog (Hh) signaling (Park et al., 2003).

Syndecan-1 (Sdc1) is a transmembrane HSPG highly enriched in the cortical VZ. Knockdown of *Sdc1* in the developing mouse cortex led to a reduction in NEC proliferation and premature differentiation, accompanied by a reduction in ß-catenin. This suggests a possible implication of Sdc1 in regulating Wnt signaling (Wang et al., 2012; Figure 1). Another subclass of proteoglycan that plays a prominent role in NEC proliferation is chondroitin sulfate proteoglycans (CSPGs), which include the Lectican family (Brevican, Neurocan, Versican, and Aggrecan), Phosphacan, CD44 and the transmembrane component NG2 (Maeda, 2015). Previous studies have shown that depletion of CSPGs in mouse neurospheres *in vitro*, by means of the CSPG degrading enzyme Chondroitinase ABC, leads to a decrease in proliferation of NECs (Sirko et al., 2007). Intriguingly, a similar treatment with Chondroitinase ABC of rat neurospheres increased NEC proliferation and differentiation, indicating some functional divergence in this respect across species (Gu et al., 2009).

Laminins are a major class of ECM components with a role in cortical progenitor proliferation. Laminins are trimeric proteins composed of alpha, beta, and gamma subunits. They are expressed at high levels in stem cell niches like the VZ and SVZ, and are a major component of the VZ’s apical surface (Lathia et al., 2007; Hall et al., 2008; Nirwane and Yao, 2019). Laminins exert their function by binding to Integrin and non-Integrin receptors, which transduce the Laminin signal in and out of the cell (Nirwane and Yao, 2019). *In vitro* studies illustrate that Laminin has an effect on expansion, maintenance and differentiation of mouse and human cortical progenitor cells (Drago et al., 1991; Kearns et al., 2003; Flanagan et al., 2007). Interestingly, enhanced expression of *Integrin-β1* in NECs of chick embryos led to two very distinct phenomena (Long et al., 2016). On the one hand, the generation of a population of cells that resemble subapical progenitors (SAPs) described in mouse (Pilz et al., 2013), dividing in the VZ away from the apical surface and producing IPCs. On the other hand, a non-cell autonomous effect where non-Integrin expressing cells undergo greater levels of neurogenesis driven by Wnt signaling and an increase in *Decorin* expression (Long et al., 2016). Because *Decorin* is only expressed in the OSVZ of the Human cortex (Fietz et al., 2012), this result further supports the notion that the ECM was key in the evolution of the mammalian cortex by enhancing the proliferation of progenitor cells and promoting cortical expansion and folding. So the next question regarding Laminins is: ¿how is their expression controlled during cortical development? A recent study reports that knock out of *Sox9* in the developing ferret cortex leads to a reduction in the proliferation of IPCs and bRGCs in the OSVZ. Conversely, conditional overexpression of *Sox9* in the embryonic mouse cortex leads to an increase in the proliferation of BPs, increased cell cycle re-entry and premature gliogenesis (Figure 1). In the long term, *Sox9* overexpression in mouse leads to an increase in the production of upper layer neurons, a hallmark of evolutionary cortical expansion. Importantly, *Sox9* overexpression in mouse cortex was accompanied by increased expression of ECM components, where Laminin 211 was the key in promoting BP proliferation (Güven et al., 2020).

Extracellular matrix components also influence the INM of NECs and aRGCs. Zebrafish *tab* mutants (analogue of *Laminin γ1*) exhibit abnormal INM in the neural tube, with nuclei entering mitosis prior to reaching the apical domain (Tsuda et al., 2010). Similarly, blockade of the β1-Integrin receptor in the VZ leads to detachment of aRGCs and affects INM and the cleavage plane of VZ progenitor cells (Figure 1; Lathia et al., 2007; Loulier et al., 2009). These studies confirm the key and evolutionarily conserved influence of Laminins and their receptors on progenitor proliferation and cortical development.

The basement membrane, produced by the meningeal membranes, is crucial for the survival of RGCs. Loss of *Integrin-β1* in aRGCs of the developing mouse cortex leads to the detachment of their end feet, followed by apoptosis. This detachment is recapitulated by surgical removal of the meninges, and in mice lacking *Laminin α2* and *4* in their basement membrane (Figure 1; Radakovits et al., 2009). Furthermore, mutant mice with disrupted meningeal development exhibit an expansion of NECs in detriment of IPC production and neurogenesis (Siegenthaler et al., 2009). This phenotype was rescued with retinoic acid (RA) treatment, showing the importance of the factors secreted from the meninges for propagating a normal neurogenesis (Siegenthaler et al., 2009).

The concept that the self-renewal capacity of cortical progenitors is the driving force for cortical expansion during evolution, where gyrencephalic species have a larger capital of NECs underlying the generation of more aRGCs, IPs and bRGCs, and subsequently more neurons, has been supported experimentally (Florio and Huttner, 2014; Fernandez et al., 2016). Integrin αvβ3 is expressed at particularly high levels in human OSVZ, where highly proliferative bRGCs are abundant. Inhibition of Integrin αvβ3 signaling in species endowed with abundant bRGCs, including human and ferret, decreases proliferation of bRGCs in OSVZ (Fietz et al., 2010; Reillo et al., 2011). Concomitantly, activation of the Integrin αvβ3 receptor in mouse cortex leads to increased proliferation and cell cycle re-entry of IPs (Stenzel et al., 2014). Altogether, this strongly supports the notion that Integrin modulation of BPs plays an important role in cortical expansion, and that changes in ECM composition during mammalian evolution contributed critically to define the size and complexity of the cerebral cortex, including progenitor cell proliferation, neurogenesis and gliogenesis (Rash et al., 2019).

## ECM in Cell Migration

Extracellular matrix molecules are also involved in regulating neuronal migration during cortical development (Franco and Müller, 2011; Franco et al., 2011). Excitatory cortical neurons travel radially from their place of birth in the germinal layers to their final destination in the CP, in a process known as radial migration (Rakic, 1972; Sidman and Rakic, 1973). In this process, neurons interact intimately with the basal process of aRGCs, known as radial glial fiber, which serves as guide and physical substrate for neuronal migration (Rakic, 1972; Sidman and Rakic, 1973). Thus, radial neuron migration depends on the integrity of RGCs, the actual movement of neurons, and the interaction between the two. Defects in neuron radial migration usually involve delayed or excessive migration, and lead to neuronal miss positioning and disorganization of cortical layers, direct causes of malformation of cortical development (Fernandez et al., 2016). Classically, studies of neuron radial migration have focused on intrinsic or cell-autonomous functions of candidate genes. However, radial neuron migration is also influenced by multiple non-cell autonomous signals, ranging from diffusible molecules to ECM proteins, and cell-cell interactions. This section mainly focuses on the role of ECM components as primary non-cell autonomous factors that affect radial neuron migration.

### Preservation of RGCs and the Basement Membrane

**FIGURE 2:**
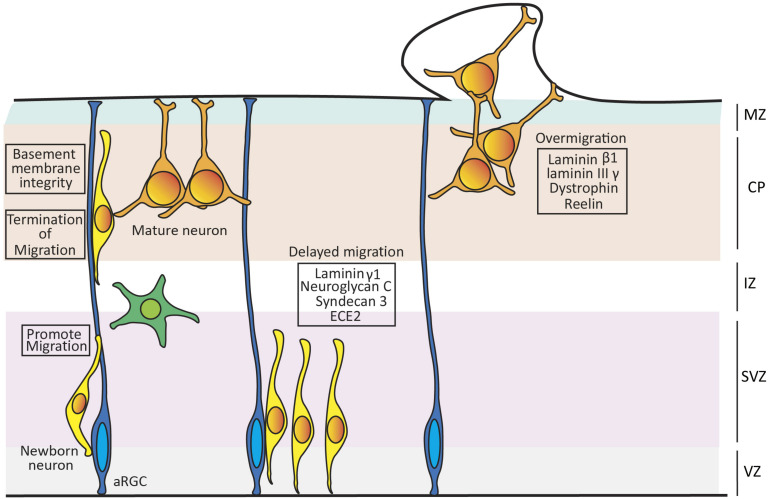
Role of extracellular matrix (ECM) on neuronal migration. Schema showing the role of ECM components on promoting the migration, termination of migration and maintenance of the basement membrane integrity in mouse developing cortex. Loss of Laminin γ1, Neuroglycan C, Syndecan 3, and overexpressing ECE2 leads to delay in migration, while loss of Laminin β1, Laminin IIIγ, Dystrophin or Reelin leads to overmigration of neurons and breaching of the basement membrane.

Radial neuron migration in the cerebral cortex depends on the integrity of RGCs, including the attachment of their basal process to the basement membrane, where ECM components are highly expressed. Laminins are critical for the structural integrity of the basement membrane, and patients with mutations in *Laminin beta-1* (*LAMB1*) develop cobblestone-lissencephaly. This is a neuronal migration disorder characterized by the breaching of the basement membrane, causing the detachment of the basal end-feet of aRGCs followed by the over migration of neurons, the loss of cortex folding and the acquisition of a bulgy appearance of the cortical surface (Timpl and Rohde, 1979; Radmanesh et al., 2013). Similarly, mutant mice deficient in *Laminin γ1III4* and *Perlecan* have severe defects on basement membrane integrity and neuron migration (Haubst et al., 2006), developing neuronal ectopias typical of cortical cobblestone (Figure 2).

Dystroglycan is another ECM component with an important role in neuron migration. This is a glycoprotein key in the dystrophin glycoprotein complex, which binds to α-Dystroglycan, a primary target for O-glycosylation. The Dystrophin glycoprotein complex is important for maintaining the integrity of the basement membrane by ensuring the attachment of the RGC end feet to the pial surface. Patients with genetic mutations resulting in hypoglycosylation of α-Dystroglycan display over-migration abnormalities and other malformations of cortical development (van Reeuwijk et al., 2005). This phenotype is mimicked in *Dag1* mutant mice, where RGCs fibers are truncated and the basement membrane is frequently breached, invaded by multiple cell types forming heterotopias (Figure 2; Myshrall et al., 2012).

The integrity of RGCs is also impaired upon the loss of the proteoglycan Syndecan-3 (Hienola et al., 2006) and of Endothelin Converting Enzyme 2 (ECE2; Buchsbaum et al., 2020). Both absence and overexpression of ECE2 in developing mouse embryos and human cerebral organoids lead to apical-basal detachment of RGCs and impaired radial neuron migration, resulting in the ectopic accumulation of neurons within the VZ. These features are typical of periventricular nodular heterotopia (PNH), a cortical malformation formed by clusters of cortical neurons that fail to undergo radial migration properly and accumulate next to the ventricular surface. Proteomic studies analyzing ECE2 mutant human cerebral organoids reveal a significant down regulation of ECM components such as *Laminin*, *Lumican* and six different collagens. These findings highlight the role of ECE2 in regulating the expression of ECM components that are important for normal neuron migration and cortical development (Figure 2; Buchsbaum et al., 2020).

### Regulation of Neuron Movement

The role of ECM in cortical lamination also extends to a direct influence on migrating neurons. Reelin (Reln) is among the most studied, and yet most poorly understood, ECM molecules. Throughout cortical development, Reln is secreted by Cajal-Retzius (CR) cells in the marginal zone (D’Arcangelo et al., 1995; Alcantara et al., 1998). Reln binds to the VLDLR and/or ApoER2 lipoprotein receptors of target cells, driving the tyrosine phosphorylation of the adaptor protein Dab1 (Rice and Curran, 2001). Reln has been proposed to be a stop signal that instructs the end of radial migration to each new wave of cortical neurons, thus directly organizing the formation of cortical layers in an inside-out manner (older neurons occupy deep layers, newer neurons occupy superficial layers). Mutation of *RELN* leads to Norman-Roberts lissencephaly in humans (Hong et al., 2000) and to the *reeler* phenotype in mice (D’Arcangelo et al., 1995). Both human and mouse mutations disrupt cortical neuron migration, which in *reeler* mice is accentuated by the massive invasion of ectopic neurons into the marginal zone. This led to the suggestion that Reln acts as a “stop” signal to terminate neuronal migration at the cortical marginal zone (Figure 2; Curran and D’Arcangelo, 1998; Dulabon et al., 2000; Rice and Curran, 2001). CR cells and Reln have also been shown to be required for maintenance of the integrity of radial glia fibers in mouse (Super et al., 2000; Hartfuss et al., 2003), but this remains under debate as it seems not to be the case in ferret (Schaefer and Juliano, 2008). The sequence of Reln protein is conserved across more than 104 species (Manoharan et al., 2015), and the levels/patterns of expression of *Reln* and *Dab1* during cortical development in turtle, lizard, chicken and mouse are well corresponded with their respective laminar organization. In contrast to the subpial expression of *Reln* in mammals, in lizards it is expressed in a subcortical layer and cortical neurons are positioned in an inverted, outside-in manner. This suggests functional conservation of this extracellular protein in neuronal migration across amniotes. Its relevance in the well-defined laminar organization of the CP in mammals and lizards, as opposed to non-laminar in birds, is considered an example of homoplasy by convergent evolution (Bar et al., 2000).

Malformations of cortical development are also caused by delayed neuronal migration (Ross and Walsh, 2001). Targeted disruption of *Laminin γ1* expression in the cerebral cortex disrupts Integrin and Akt/Gsk-3β signaling, which impairs neuronal migration without affecting cell proliferation and neuronal cell death. The absence of Laminin γ1 – AKT signaling hinders the arrival of migrating neurons to the marginal zone and leads to defective cortical lamination (Figure 2; Chen et al., 2009). Neuroglycan C is a member of the family of CSPGs and a downstream interactor of PHF6, an X-linked protein mutated in the intellectual disability disorder Börjeson–Forssman–Lehmann. Loss of Neuroglycan C in mouse embryos leads to radial migration failure during cortical development (Figure 2; Zhang et al., 2013). The functional side chains of CSPGs possess a sulphated structure generated by a family of sulphotransferases, several of which are expressed during cortical development. Several sulphotransferases have been shown to play central roles in neuronal migration, by *in utero* electroporation of loss-of-function short hairpin RNAs. Following this manipulation, neuronal migration is blocked at the multipolar-to-bipolar transition but not at the level of RGCs, suggesting that the specific sulphated side chains play an important role during radial migration (Akita et al., 2008; Ishii and Maeda, 2008). Altogether, it is clear that the ECM is involved in controlling many aspects of cortical neuronal migration, and that this is largely conserved across phylogeny, further supporting the importance of the ECM on the expansion and folding of the cerebral cortex during evolution.

## ECM in Cerebral Cortex Folding

As mentioned above, transcriptomic studies have demonstrated that expression of ECM components is very different between cortical layers and species, supporting a process of cortical expansion and folding via progenitor cell proliferation and neuron migration. The ECM also defines the stiffness and biomechanical properties of the developing cortex, thus additionally influencing its folding. Accordingly, changes in ECM composition during mammalian evolution may have dictated the occurrence, degree and pattern of cortex folding across phylogeny (Llinares-Benadero and Borrell, 2019).

### ECM in Cortical Expansion

The mechanisms responsible for folding of the mammalian cerebral cortex have been under debate for many years. An early attractive hypothesis was that animals with large brains have folded cortices because they undergo a disproportionate expansion of the outer cortical surface (gray matter, composed of neuron) in comparison to the inner part (white matter, composed of axons and glial cells), and this leads to folding of the cortex. Notable exceptions to this trend are represented by the American beaver and the Florida manatee, which have a smooth cortex but brain size similar to other species with a highly folded cortex, such as the chimpanzee (Welker, 1990). A refined version of this hypothesis proposes that cortex folding results from the differential expansion of the upper neuronal layers in comparison to deep cortical layers (Armstrong et al., 1991). The relative expansion of upper layers has been proposed to result from increases in BP abundance and the formation of the OSVZ (Smart et al., 2002; Kriegstein et al., 2006; Reillo et al., 2011; Borrell and Reillo, 2012). In combination with differential neurogenesis, the tangential dispersion of radially migrating neurons in gyrencephalic species is thought to significantly contribute to the expansion of cortical surface and the formation of folds (Borrell, 2018; Llinares-Benadero and Borrell, 2019).

As discussed above, the ECM is a very important factor in the regulation of cortical progenitor cell proliferation, and recent studies support that it is also important in cortex folding. Patients with mutations in *RELN* (see above) display abnormal neuronal migration and axonal connectivity, and in the long term resulting in lissencephaly (loss of cortical folds; Hong et al., 2000). The importance of proper neuron migration for cortical gyrification has been recently highlighted with the analysis of mice mutant for Flrt proteins. Flrts are a family of cell adhesion transmembrane proteins rich in Fibronectin and Leucine repeats, which are involved in the radial migration of cortical neurons. The analysis of mice double mutant for *Flrt1/3* revealed the formation of *bona fide* cortical folds and fissures in the otherwise smooth mouse cortex (del Toro et al., 2017). This phenotype emerges from an imbalance in adhesion-repulsion forces in migrating neurons. Importantly, these experimental results are validated by observations in the normally folded cortex of ferrets, where *Flrt1* and *Flrt3* are expressed at much lower levels in migrating neurons of cortical fissures than folds (De Juan Romero et al., 2015; del Toro et al., 2017).

### Influence of the ECM on the Mechanical Properties of Cortex During Folding

Folding of the cerebral cortex is ultimately a physical process of deformation of developing neural tissue (Kroenke and Bayly, 2018). Cortical folding has been described as a mechanism where the differential expansion rate between upper and lower cortical layers leads to elastic instability (Richman et al., 1975; Bayly et al., 2014). Experimental testing with hydrogel models has been fundamental to our understanding of this process beyond mathematical models. Hydrogel models are composed of an inner core hydrogel covered with an outer layer of second hydrogel with similar or different physical properties (elasticity, resistance, etc.). When subject to expansion, these compound gel models sustain significant and measurable elastic instability and compression. The use of these models has demonstrated that when the outer layer swells (grows) faster than the inner core, this results in material strain and compression, which is released by buckling and the formation of seeming folds and fissures (Tallinen et al., 2014). For greater realism, three-dimensional hydrogel models have been designed with the shape of a mid-gestational human embryo brain, and then the differential expansion of the bi-layered hydrogel results in the formation of folds and fissures mimicking the adult human brain (Tallinen et al., 2016).

The above studies and related transcriptomic analyses (Sheppard et al., 1991; Fietz et al., 2012) suggest that the ECM regulates cortical folding not only by affecting progenitor cell proliferation and neuron migration, but also by contributing to define the mechanical properties of the developing cortex. A seminal study by Long and colleagues used living slices of embryonic human cortex cultured *in vitro* to demonstrate the critical role of the ECM on cortex folding (Long et al., 2018). Slices of human fetal neocortex in culture were treated with a cocktail of ECM components (HAPLN1, Lumican, and Collagen I), which induced the ultra-rapid folding of the cortical surface, not occurring in untreated slices. Related to an increase in tissue stiffness, this folding was accompanied by an increase in expression of HA and its receptor (CD168) in the CP, followed by ERK signaling activation. Intriguingly, this ECM cocktail did not induce folding by promoting progenitor proliferation or neuronal migration, but by decreasing cell density at the CP. This was recapitulated in untreated slices from older fetuses, supporting that this combination of ECM components increases stiffness and induces folding by the same physiological mechanism as nascent folds that develop at later stages in the non-manipulated human embryo (Long et al., 2018).

The advent of cerebral organoids has become an additional alternative to study and understand cortical folding, by physical manipulation *in vitro*. An innovative organoid on-a-chip approach allows growing cerebral organoids that wrinkle and fold (Karzbrun et al., 2018). This enables to culture human cerebral organoids in millimeter-thick chambers and image them in whole mount, including the formation of folds. Under these conditions, organoids developed from hiPSCs from lissencephalic patients, mutant for *LIS1*, wrinkle significantly less than control organoids from healthy donors. Transcriptomic analyses of these mutant organoids has revealed a significant downregulation of ECM and cytoskeletal genes, suggesting that the underlying cause of this deficit in cortical folding is a pathological softening of the cytoskeleton. Unfortunately, cortical folding of on-chip organoids is due to contraction of the VZ and expansion of the progenitor cell nucleus (Karzbrun et al., 2018), which completely differs from the expanded basal germinal zones and increased neurogenesis observed in animal models (Reillo et al., 2011; Heide et al., 2018; Karlinski and Reiner, 2018; Karzbrun et al., 2018). Nonetheless, these results support the relevance of the ECM in maintaining the tissue contractility and stiffness that induce cortex folding (Karlinski and Reiner, 2018; Karzbrun et al., 2018).

The balance between softness and stiffness in the CNS microenvironment is also a key factor in fate determination. Mounting evidence demonstrates that the mechanical properties of tissue microenvironment exerted by ECM components, including stiffness or viscoelasticity, play a significant role in cell fate determination, dictating the output of cellular lineages from differentiation to proliferation or apoptosis (Holle et al., 2018). For example, microenvironments as soft as brain tissue promote mesenchymal stem cells to adopt a neuronal lineage, whereas stiffer microenvironments promote the same cells to enter myogenic differentiation (Engler et al., 2006). Analyses of the stiffness of the developing mouse cortex using atomic force microscopy (AFM) have shown that VZ and SVZ gradually increase in stiffness during development, while the neuron-rich CP increases in stiffness only until E16.5, decreasing by E18.5. Stiffness of the CP is due not only to neurons, which are stiffer than other cells in the cortex, but also to changes in the composition of the ECM (Iwashita et al., 2014). Indeed, differences in ECM composition along the human cortical surface, causing variations in tissue stiffness, have been proposed as a mechanism contributing to cortex folding (Long et al., 2018; Wianny et al., 2018).

## Evolution of ECM Components and the Evolution of Cortical Folding

Recent progress in neuroimaging techniques and neuroanatomy are providing major insights into fundamental differences in cortical organization across phylogeny. Using multiple approaches to compare cortical folding, parcellation and neural connectivity in mouse, marmoset, macaque and human, David Van Essen and colleagues have revealed dramatic differences in the total number and arrangement of cortical areas (Van Essen et al., 2019). In this study, they also report that cortical folding patterns vary dramatically across species, and that individual variability in cortical folding increases with cortical surface area. In line with this evidence, recent hypotheses propose that the sophistication of cortical folding and expansion in development and evolution may be attributed to both cell autonomous mechanisms (i.e., increased progenitor cell proliferation) and non-cell autonomous mechanisms (i.e., ECM composition) known to impinge on the former (Fietz et al., 2010; Güven et al., 2020). The notion that the evolution of ECM components may have significantly contributed to the evolution of cortical folding is directly supported by the effects of ECM treatment on folding of cortical slices in culture (Long et al., 2018). Ectopic administration of ECM molecules (HAPLN1, Lumican and Collagen I) caused the folding of living cortical slices from human embryos, but not from ferrets or mice, although it did cause changes in tissue stiffness. This different response suggests that the ECM and signaling pathways that induce gyrification in humans are different from those with a similar role in ferret, as shown in Table 1. These findings highlight human specific ECM components as a game changer in mechanical and signaling processes during cortical folding (Wianny et al., 2018). Interestingly, Cromar et al. (2014) showed that ECM proteins underwent domain gain that occurs exclusively at the divergence of primates from other mammals. In agreement with this, primate-specific miRNAs regulating the expression of ECM genes are differentially expressed in CP and germinal zones in primates (Arcila et al., 2014). Taken together, this indicates the existence of evolutionary changes in the regulation of expression of ECM components, and supports the notion that the ECM contributes to regulate cortex size and folding (Fietz et al., 2012; Florio et al., 2017; Long et al., 2018).

A close inspection of the spatial and temporal patterns of expression of ECM components and cell adhesion molecules in the developing cerebral cortex highlights potential mechanisms evolved to induce cortical folding. As mentioned, *Flrt1/3* are expressed homogeneously and at high levels in the developing mouse cortex but not in ferret, where domains of medium and low expression alternate, correlating with the folding pattern. Interestingly, the loss of *Flrt1/3* in the mouse smooth cortex alters the adhesion-repulsion balance between migrating neurons thus promoting their tangential dispersion, leading to the formation of fissures and folds. This mimicks the native situation found in human and ferret, therefore emphasizing the importance of repression of *Flrt1/3* in the evolution of cortex folding (del Toro et al., 2017; Llinares-Benadero and Borrell, 2019).

The relevance of neuronal migration in the formation of cortical folds is further supported by comparative analyses in mouse and ferret (Gertz and Kriegstein, 2015; Martínez-Martínez et al., 2019). Whereas in mouse cortex radial neuron migration takes place in rather rectilinear trajectories, cortical neurons in ferret display much more tortuous and complex behaviors (Gertz and Kriegstein, 2015). Examination of the detailed cellular morphology and behavior demonstrates that, contrary to dogma, radially migrating cortical excitatory neurons extend a leading process that is frequently branched under normal physiological conditions, both in mouse and ferret (Martínez-Martínez et al., 2019). The frequency and degree of branching of this leading process are significantly greater in the gyrencephalic ferret than the lissencephalic mouse. We have proposed that this difference has a profound influence on the tangential dispersion of neurons migrating radially and, consequently, on cortical folding. Differences in branching between species may stem from differences in the expression profile of ECM and cell adhesion molecules (Fietz et al., 2012; Reillo et al., 2017).

In addition to the known and potential direct effects of ECM on cortex expansion and folding, a recent study in the developing ferret identified multiple cellular elements that may act as non-cell autonomous or “extrinsic” elements affecting cortical progenitor behavior and fate in different ways (Reillo et al., 2017). For example, axonal fiber tracts and tangentially migrating neurons with a marked laminar organization are proposed to be prominent sources of instructive signals onto cortical progenitor cells and radially migrating neurons. These extrinsic elements change quite dynamically during development, so their relevance on cortex development/folding are proposed to be also dynamic. This highlights the role that different combinations of ECM components and cell adhesion molecules may play in creating a complex laminar code of extrinsic influences, that modulate cortical development and folding in a selective manner (Nowakowski et al., 2017; Reillo et al., 2017).

## Conclusion and Future Perspectives

The ECM is best known for providing structural support to cells and tissues. However, the burst of transcriptomic studies over the past few years has identified ECM components as prime candidates in controlling cerebral cortex development, expansion and folding, and the evolution of these features. A number of studies have shown the central importance of the ECM in regulating cortical progenitor proliferation and basal progenitor amplification, the basis for increased neurogenesis, expansion and folding. Other ECM molecules regulate neuron migration or define the stiffness of tissue, with profound implications in cell fate determination and cortex folding. Some of these functions are highly conserved across phylogeny, while others exert their function in a species-specific manner. Accordingly, functionally relevant interspecies differences in ECM composition suggest its co-evolution with the cortical phenotype.

New tools and technologies continuously provide unprecedented opportunities to increase our understanding of the ECM and its roles in brain development. Single cell RNA sequencing now offers the unique opportunity to carefully examine differences in ECM expression profiles across progenitor cell populations and their lineages, and the impact of the ECM on transcriptional programs critical during cortical development. This may then allow identifying ECM signaling pathways implicated in the evolution and folding of the neocortex. A focus on the ECM is a promising strategy in the quest to reach a unified understanding of molecular mechanisms of cortical evolution and folding.

## Author Contributions

SA created the figures. SA and VB wrote the manuscript. Both authors contributed to the article and approved the submitted version.

## Conflict of Interest

The authors declare that the research was conducted in the absence of any commercial or financial relationships that could be construed as a potential conflict of interest.
